# Effect of an *Ulva lactuca* Extract on Systemic Antioxidant and Inflammatory Biomarkers in Healthy Adults: A Randomized, Double-Blind, Placebo-Controlled Trial

**DOI:** 10.3390/nu18142244

**Published:** 2026-07-09

**Authors:** Ralf Jäger, Hendrik Luesch, Martin Purpura, Ashok Godavarthi, Sebastian T. Balcombe, Ecaterina Vasenina

**Affiliations:** 1 Increnovo LLC, Whitefish Bay, WI 53217, USA; 2 Department of Medicinal Chemistry, Center for Natural Products, Drug Discovery and Development (CNPD3), College of Pharmacy, University of Florida, Gainesville, FL 32610, USA; 3 Program in Cancer and Stem Cell Biology, Duke-NUS Medical School, Singapore 169857, Singapore; 4 Radiant Research Services Pvt. Ltd., Bangalore 560058, India; 5 BluGen LLC, Boca Raton, FL 33432, USA; 6 Human Performance Laboratory, Department of Health Sciences and Human Performance, The University of Tampa, Tampa, FL 33606, USA

**Keywords:** sea lettuce, ulvan, oxidative stress, glutathione, glutathione peroxidase, catalase, interleukin-6, *C*-reactive protein, Nrf2, marine bioactives

## Abstract

**Background:** *Ulva lactuca* (sea lettuce) is a green macroalgae rich in sulfated polysaccharides (ulvans), polyphenols, and pigments with documented antioxidant and anti-inflammatory activity in preclinical models. Translational human data remain limited. **Methods:** Twenty healthy adults (20–35 years; BMI 18.5–30 kg/m^2^) were enrolled in a single-center, randomized, double-blind, placebo-controlled, parallel-group trial (CTRI/2025/11/097278) and assigned 1:1 to receive 300 mg of an *U. lactuca* extract or matched placebo once daily for 28 days. Antioxidant biomarkers (GSH, MDA, catalase, GPx-1, thioredoxin) and inflammatory biomarkers (CRP, IL-6, IL-1β, IL-11, PPAR-γ) were measured at Day 0 and Day 28. Safety was assessed throughout. **Results:** Eighteen of 20 participants completed the study. Compared with placebo, *U. lactuca* produced significant within-group improvements at Day 28 in GSH (+4.04 ng/mL), GPx-1 (+67.8 µU/mL), and catalase (+32.4 kU/L) and reductions in MDA (−6.08 nmol/mL) and thioredoxin (−1.48 ng/mL); inflammatory markers CRP (−0.91 mg/L), IL-6 (−2.97 pg/mL), IL-11 (−4.28 ± 1.88 ng/mL) and IL-1β (−8.87 pg/mL) decreased and PPAR-γ increased. **Conclusions:** In this short-duration pilot trial, daily supplementation with 300 mg of an *U. lactuca* extract was well tolerated and modulated systemic oxidative-stress and low-grade inflammatory biomarkers in healthy adults. Larger and longer studies in populations with elevated oxidative stress are warranted.

## 1. Introduction

Chronic low-grade oxidative stress and systemic inflammation are increasingly recognized as upstream contributors to a wide spectrum of non-communicable diseases, including cardiometabolic disease, neurodegeneration, sarcopenia, and accelerated biological ageing [1,2]. Even in apparently healthy adults, modest perturbations in redox balance and circulating inflammatory mediators predict long-term disease risk and functional decline [3]. Dietary and nutraceutical strategies that modulate these pathways have therefore attracted substantial scientific and consumer interest [4].

Marine algae represent a structurally diverse and largely under-exploited source of bioactive compounds [5,6]. Among them, *U. lactuca* (sea lettuce) is a fast-growing green macroalgae distributed across temperate and tropical coastal waters. It is responsible for recurrent green-tide blooms that present an environmental burden but, equally, a sustainable biomass resource harvested for various industrial and nutritional applications [7,8]. The chemical composition of *U. lactuca* is dominated by sulfated heteropolysaccharides (termed ulvans), together with polyphenols, carotenoids, and chlorophylls, as well as a lipid fraction that includes structurally unique unsaturated fatty acid derivatives [9,10]. In addition to conventional polyunsaturated fatty acids, *U. lactuca* contains electrophilic keto-type fatty acids bearing α,β-unsaturated carbonyl moieties that have been shown to activate the Nrf2–antioxidant response element (ARE) pathway and induce cytoprotective gene expression [11]. These findings suggest that the lipid fraction may contribute to the biological activity of *U. lactuca* not only through nutritional effects but also via direct modulation of redox-sensitive signaling pathways. Ulvans, in particular, have been shown in vitro and in animal models to scavenge reactive oxygen species (ROS), inhibit NF-κB-driven cytokine production, and modulate macrophage polarization [12,13,14]. Recent work has additionally demonstrated that low-molecular-weight ulvan oligosaccharides attenuate experimental colitis via suppression of pro-inflammatory cytokine cascades [15], while selenium nanoparticles decorated with *U. lactuca* polysaccharides inhibit NF-κB-mediated hyper-inflammation in macrophage models [16].

Despite this preclinical foundation, controlled human data on *U. lactuca* remain limited. Allaert and colleagues reported favorable mood-related outcomes in a randomized double-blind trial of an edible *U. lactuca* extract in volunteers with anhedonia [17]. More recently, Roach et al. conducted two randomized placebo-controlled trials of SXRG84, a sulfated xylorhamnoglucuronan derived from *Ulva* sp. 84, in overweight and obese adults, reporting reductions in non-HDL cholesterol, CRP, and several pro-inflammatory cytokines (IFN-γ, IL-1β, TNF-α) following six weeks of supplementation at 2–4 g/day [18,19]. However, direct biochemical evidence that *U. lactuca* supplementation modulates systemic antioxidant and inflammatory biomarkers in healthy, non-obese adults has not been reported.

To address this gap, the present study evaluated the effects of short-term oral supplementation with *U. lactuca* extract on systemic biomarkers of oxidative stress and inflammation in healthy adults. In a randomized, double-blind, placebo-controlled design, participants received a standardized *U. lactuca* extract or placebo daily for 28 days. We hypothesized that *U. lactuca* supplementation would improve antioxidant status, reflected by favorable changes in glutathione, glutathione peroxidase-1, catalase, and malondialdehyde, while also attenuating circulating inflammatory biomarkers, including *C*-reactive protein, interleukin-6, and interleukin-1β, compared with placebo.

## 2. Materials and Methods

### 2.1. Trial Design and Oversight

This was a single-center, randomized, double-blind, placebo-controlled, parallel-group clinical trial conducted at Sri Venkateshwara Hospital (Bangalore, Karnataka, India) between 20 November 2025 and 7 January 2026. The study protocol was approved by an independent ethics committee on 25 August 2025, prospectively registered with the Clinical Trials Registry—India (CTRI/2025/11/097278), and conducted in accordance with the Declaration of Helsinki, ICH–Good Clinical Practice, and applicable Indian regulatory requirements. The full clinical study report was prepared in accordance with ICH E3. Written informed consent was obtained from every participant prior to any study procedure.

### 2.2. Participants

Eligible participants were healthy male and female adults aged 20–35 years with a body-mass index (BMI) of 18.5–30 kg/m^2^ and a self-reported habit of regular physical activity (cycling, running, elliptical training, push-ups, or swimming) on at least three days per week. Participants agreed to refrain from prescription and over-the-counter medications, vitamins, mineral supplements, and any alternative-medicine preparation for the duration of the study, and reported no known allergy to algae or shellfish and no history of cardiovascular disease, diabetes, or cancer.

Key exclusion criteria were pregnancy or breast-feeding; hypersensitivity to algae or shellfish; pre-existing severe systemic disease requiring chronic medication; current use of medications likely to interact with algae extract; any history of solid or hematologic malignancy or carcinoma in situ; and participation in another interventional clinical trial within the previous month.

### 2.3. Randomization and Blinding

Eligible participants were randomized 1:1 to active or placebo using a computer-generated permuted-block sequence (block size = 2) prepared by an independent statistician at Radiant Research Services Pvt. Ltd. and held in sealed, sequentially numbered envelopes. Investigational and placebo capsules were identical in appearance, weight, and packaging. Participants, investigators, site staff, laboratory personnel, and the data analyst were blinded to treatment allocation. The randomization code was broken only after database lock, by the designated biostatistician, for the purpose of final statistical analysis.

### 2.4. Investigational Product and Intervention

The investigational product was a proprietary *U. lactuca* extract (Algevity^®^, BluGen LLC, Boca Raton, FL, USA) supplied in 300 mg capsules and produced using supercritical CO_2_ extraction, a process designed to enrich lipophilic constituents, including unsaturated fatty acids. The content of the investigational product was verified by independent third-party analysis (Interfield Laboratories, Kochi, India). The extract contained 8% total polysaccharides and 3% total lipids, including the keto-unsaturated fatty acid derivatives 7(E)-9-keto-octadec-7-enoic acid, 7(E)-9-keto-hexadec-7-enoic acid, and 7(E)-9-keto-octadec-7-enamide, which have previously been identified in *Ulva lactuca* and reported as activators of the Nrf2–ARE pathway [11]. In addition, product identity was verified using FTIR, HPTLC, HRMS, and GC/MS fingerprint analyses. Placebo capsules contained 300 mg of inert excipients and were matched for appearance and organoleptic properties. Participants in both arms ingested one capsule once daily, orally, for 28 consecutive days. Study visits were scheduled at screening (Day −5), Day 0 (baseline/first dose), Day 14, and Day 28 (end of treatment). Returned capsule counts were used to verify compliance; treatment compliance was 100% in both arms in the per-protocol set. Participants were instructed to maintain their usual dietary habits and physical-activity patterns throughout the study and not to initiate any new nutritional supplements or alternative-medicine preparations during the intervention period. In addition, participants were instructed to maintain their usual physical-activity patterns and lifestyle habits throughout the study and not to initiate any new exercise programs during the intervention period.

### 2.5. Outcomes

Primary outcomes were safety and tolerability assessed via adverse events, vital signs, and a complete hematology and clinical-chemistry panel at baseline, Day 14, and Day 28. Antioxidant and inflammatory biomarkers were prospectively specified for assessment at baseline (Day 0) and end-of-treatment (Day 28) only. Primary efficacy outcomes were the changes from baseline to Day 28 in serum antioxidant biomarkers: reduced glutathione (GSH), malondialdehyde (MDA), catalase activity, and glutathione peroxidase-1 (GPx-1). Secondary outcomes comprised change from baseline to Day 28 in circulating inflammatory and immunomodulatory mediators: *C*-reactive protein (CRP), interleukin-6 (IL-6), interleukin-1β (IL-1β), interleukin-11 (IL-11), peroxisome proliferator-activated receptor gamma (PPAR-γ), and thioredoxin (TXN).

### 2.6. Laboratory Methods

Venous blood samples were collected after an overnight fast at each scheduled visit. Serum and plasma aliquots were stored at −80 °C until batched analysis to minimize inter-assay variability. Antioxidant biomarkers were quantified using validated colorimetric assays (GSH [manufacturer: Bioassay Technology Laboratory (BT Labs, Shanghai, China), catalogue number: Ea0142hu, kit range: 0.1–40 ng/mL], MDA [BT Labs, E1371hu, 0.2–70 nmol/mL], catalase activity [BT Labs, E3053hu, 2–600 kU/L]) and an enzyme-coupled assay for GPx-1 [BT Labs, E3921hu, 3–900 μU/mL]. Inflammatory biomarkers (CRP [BT Labs, E1798hu, 0.02–6 mg/L], IL-6 [BT Labs, E0090hu, 2–600 pg/mL], IL-1β [BT Labs, E0143hu, 20–6000 pg/mL], IL-11 [BT Labs, E0101hu, 0.5–300 ng/mL], PPAR-γ [BT Labs, E1511hu, 30–9000 ng/L], TXN [ELK Biotechnology Co., Ltd. (Wuhan, Hubei, China), Elk10348, 0.32–20 ng/mL]) were quantified by commercial enzyme-linked immunosorbent assay (ELISA) according to the manufacturers’ instructions. All assays were performed in duplicate by personnel to treatment allocation, participant identity, and visit sequence, and reported values represent the mean of duplicate determinations.

### 2.7. Sample Size

As an exploratory pilot study designed to inform effect sizes for subsequent confirmatory trials, the sample size of 20 participants (10 per arm) was selected on feasibility grounds rather than on a formal power calculation, in line with current methodological guidance for early-phase nutraceutical trials [20].

### 2.8. Statistical Analysis

Analyses were conducted on two pre-specified populations: the intention-to-treat (ITT) population, which included all randomized participants who received at least one dose of study medication (*n* = 20), and the per-protocol set (PPS), which included participants who completed the study without major protocol deviations (*n* = 18). For the ITT analyses, missing Day 28 values were imputed using the Last Observation Carried Forward (LOCF) method. The primary efficacy analyses presented in the manuscript were performed on the ITT population. PPS analysis included only participants with available Day 28 data and were conducted as supportive analyses. Results from the PPS analyses were directionally consistent with those observed in the ITT population. All statistical analyses were conducted according to a pre-specified Statistical Analysis Plan (SAP) that was finalized prior to treatment unblinding. As a sensitivity analysis, Mixed Model Repeated Measures (MMRM) models adjusted for age and sex were also evaluated and yielded conclusions that were generally consistent with the primary ITT analyses.

Continuous variables are summarized as mean ± standard deviation (SD), median, and 95% confidence interval. Within-group changes from Day 0 to Day 28 were tested using a paired Student’s *t*-test or, where the assumption of normality was not satisfied (Shapiro–Wilk *p* < 0.05), the Wilcoxon signed-rank test. Between-group comparisons at each visit and between-group comparisons of change scores were tested using an independent-samples *t*-test or, where appropriate, the Mann–Whitney U test. Categorical variables were compared using Fisher’s exact test. Two-sided *p* < 0.05 was considered statistically significant. Given the exploratory nature of the trial, no adjustment for multiplicity was applied. Analyses were performed using IBM SPSS 25.0 and SAS v9.4 (SAS Institute, Cary, NC, USA).

### 2.9. Safety Assessment and Causality

Adverse events (AEs) were collected by spontaneous report and direct questioning at each visit, classified by MedDRA System Organ Class, and graded for severity according to standard criteria (mild—no intervention required; moderate—intervention required; severe—significantly disabling or life-threatening). Causality was assessed by the principal investigator using the Naranjo Adverse Drug Reaction Probability Scale [21], with a score of ≥9 categorized as definite, 5–8 as probable, 1–4 as possible, and ≤0 as doubtful (not related).

## 3. Results

### 3.1. Participant Disposition and Baseline Characteristics

**Figure 1:**
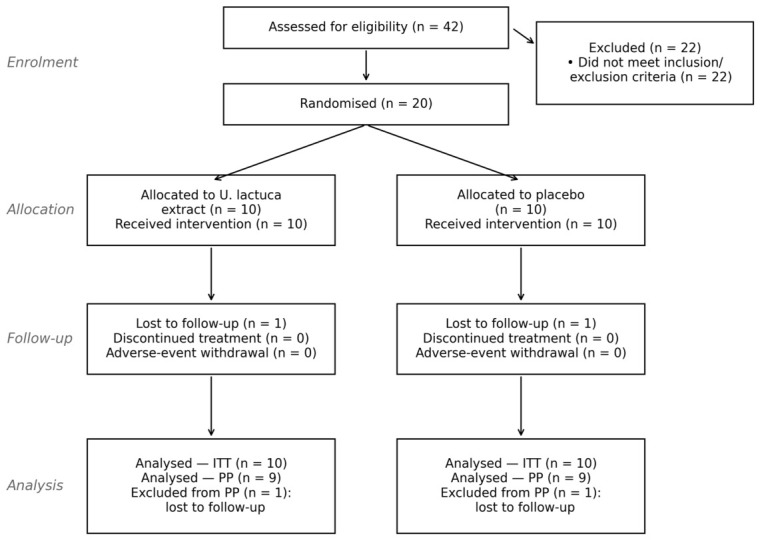
CONSORT flow diagram of participant screening, randomization, follow-up, and analysis. ITT, intention-to-treat; PP, per-protocol.

Of 42 individuals assessed for eligibility, 22 were excluded for failing to meet inclusion or exclusion criteria, and 20 were randomized in a 1:1 ratio to *U. lactuca* extract (Group ALX, *n* = 10) or placebo (Group PLB, *n* = 10) (Figure 1). Eighteen participants (90%) completed all study visits; one participant in each arm was lost to follow-up after randomization. No participant discontinued for adverse events, protocol violations, or compliance failure. Treatment compliance, verified by returned capsule count, was 100% in both arms in the per-protocol set.

Demographic and baseline anthropometric characteristics were comparable between the two arms (Table 1). Mean age was 28.7 ± 4.6 years in Group ALX and 30.8 ± 4.0 years in Group PLB; mean BMI was 23.4 ± 0.8 and 23.1 ± 1.2 kg/m^2^, respectively. The proportion of female participants was higher in Group ALX (60%) than in Group PLB (20%), but this difference was not statistically significant (Fisher’s exact *p* = 0.17). All baseline biomarker values were similar between arms (all *p* > 0.5; Table 2 and Supplementary Tables S1 and S2).

### 3.2. Effect on Systemic Antioxidant Biomarkers

**Figure 2:**
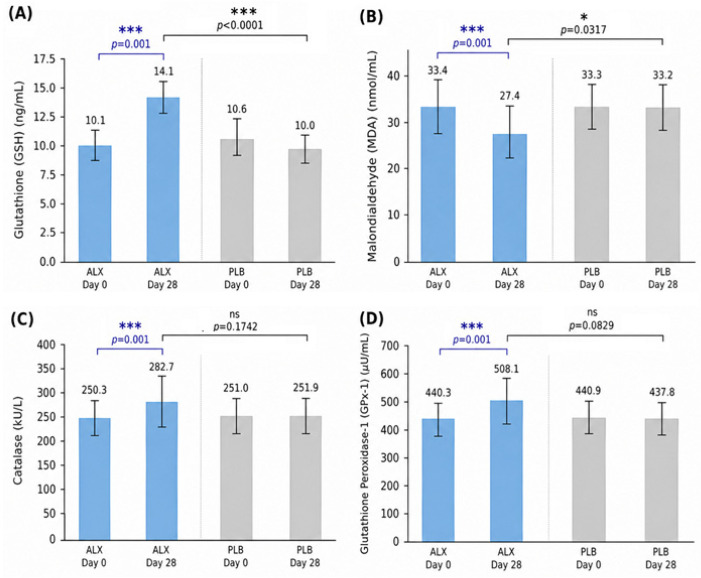
Effect of *U. lactuca* extract (ALX, blue) versus placebo (PLB, grey) on serum antioxidant biomarkers at Day 0 and Day 28 (ITT population, *n* = 10 per arm). (**A**) Reduced glutathione (GSH); (**B**) malondialdehyde (MDA); (**C**) catalase activity; (**D**) glutathione peroxidase-1 (GPx-1). Bars show mean ± SD. *p*-values shown above brackets are between-group comparisons at Day 28 (gray) or within-group comparisons (blue). *** *p* < 0.001; * *p* < 0.05; ns = not significant.

Baseline values of all four antioxidant biomarkers were similar between arms (all *p* > 0.5; Table 2). After 28 days of supplementation, Group ALX showed a marked, statistically significant within-group increase in serum GSH (+4.0 ± 1.9 ng/mL; paired-test *p* < 0.0001) that was paralleled by a small but significant decrease in placebo (−0.6 ± 0.6 ng/mL; *p* = 0.0058). The between-group difference at Day 28 and the between-group difference in change from baseline were both highly significant (both *p* < 0.0001; Figure 2A). MDA, a marker of lipid peroxidation, decreased significantly in Group ALX (−6.1 ± 2.9 nmol/mL; *p* < 0.0001) while remaining essentially unchanged in placebo (−0.1 ± 1.0 nmol/mL; *p* = 0.69), with a significant between-group difference at Day 28 (*p* = 0.0317; Figure 2B).

Catalase activity increased significantly within Group ALX (+32.4 ± 22.2 kU/L; *p* = 0.001) but was unchanged in placebo (+0.9 ± 3.49 kU/L; *p* = 0.44); the between-group difference in change from baseline was statistically significant (*p* = 0.0014), although the between-group comparison at the Day 28 visit alone did not reach significance (*p* = 0.1742; Figure 2C). GPx-1 activity rose markedly in Group ALX (+67.8 ± 27.7 µU/mL; *p* < 0.0001) versus a non-significant decrease in placebo (−3.0 ± 7.7 µU/mL; *p* = 0.25), with a significant between-group difference in change from baseline (*p* = 0.0007; Figure 2D).

### 3.3. Effect on Circulating Inflammatory Biomarkers

**Figure 3:**
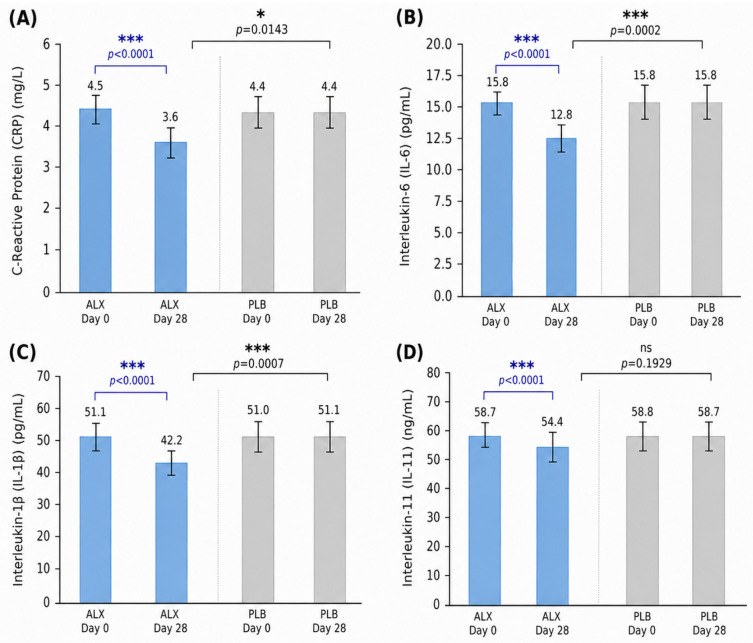
Effect of *U. lactuca* extract (ALX) versus placebo (PLB) on circulating inflammatory biomarkers at Day 0 and Day 28 (ITT, *n* = 10 per arm). (**A**) CRP; (**B**) IL-6; (**C**) IL-1β; (**D**) IL-11. *** *p* < 0.001; * *p* < 0.05; ns = not significant.

**Figure 4:**
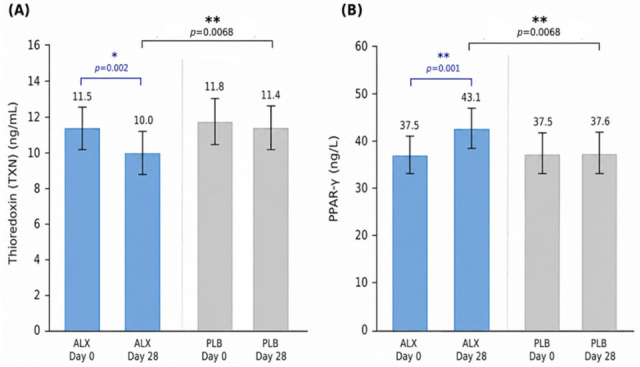
Effect of *U. lactuca* extract (ALX) versus placebo (PLB) on (**A**) thioredoxin (TXN) and (**B**) PPAR-γ at Day 0 and Day 28 (ITT, *n* = 10 per arm). ** *p* < 0.01; * *p* < 0.05.

All baseline inflammatory biomarker values were comparable between arms (Table 2). After 28 days, Group ALX exhibited statistically significant within-group reductions in CRP (−0.9 ± 0.4 mg/L; *p* < 0.0001), IL-6 (−3.0 ± 1.1 pg/mL; *p* < 0.0001) and IL-1β (−8.9 ± 3.6 pg/mL; *p* < 0.0001), and a statistically significant within-group increase in PPAR-γ (+5.7 ± 2.3 ng/L; *p* = 0.001). The between-group differences at Day 28 were significant for all four mediators (CRP *p* = 0.0143; IL-6 *p* = 0.0002; IL-1β *p* = 0.0007; PPAR-γ *p* = 0.0068; Figure 3A–C and Figure 4B). Placebo showed a small but statistically significant increase in CRP (+0.1 ± 0.1 mg/L; *p* = 0.0112) consistent with day-to-day biological variability in this analyte while remaining stable for IL-6 and IL-1β.

IL-11 declined within Group ALX (−4.3 ± 1.9 ng/mL; within-group *p* < 0.0001) with no change in placebo (−0.1 ± 0.9 ng/mL; *p* = 0.6498); however, the between-group difference at Day 28 did not reach statistical significance (*p* = 0.1929; Figure 3D). Recent evidence has shifted the understanding of IL-11 from a pleiotropic cytokine with mixed roles toward a predominantly pro-inflammatory and pro-fibrotic mediator, implicated in tissue remodeling, fibrosis, and age-related pathologies [22]. Circulating IL-11 levels have been reported to increase with aging and are associated with chronic low-grade inflammation and degenerative processes [23]. In this context, the observed reduction in IL-11 within Group ALX may suggest a potential modulation of pathways linked to inflammaging and fibrosis. However, given the absence of a statistically significant between-group difference and the evolving understanding of IL-11 biology, the clinical relevance of this finding in healthy adults remains uncertain.

Thioredoxin, a thiol-redox protein with both antioxidant and inflammatory-modulating functions, decreased significantly in Group ALX (−1.5 ± 0.7 ng/mL; *p* = 0.002) and was significantly different between arms at Day 28 (*p* = 0.0068; Figure 4A). Reduced circulating thioredoxin in this context is consistent with diminished oxidative-stress signaling [24].

### 3.4. Safety, Tolerability, and Adverse Events

A total of four adverse events were reported across the 28-day treatment period: one event of bloating in Group PLB (Visit 2; onset Day +10), and three events in Group ALX at Visit 3 (skin rash, nausea, and mild diarrhea; all with onset ≥ Day +20 from first dose). All events were transient, resolved within 3 days with standard symptomatic treatment, and did not require discontinuation of the investigational product (Table 3).

Causality was assessed for every adverse event using the Naranjo Adverse Drug Reaction Probability Scale by the principal investigator. The cumulative Naranjo score was ≤0 for every event, corresponding to a “doubtful” classification (i.e., not related to the investigational product). This assessment was based on three converging considerations: (i) the absence of a consistent temporal relationship; onset occurred ≥10 days after first dose in the placebo arm and ≥20 days after first dose in the active arm, a pattern not consistent with an acute or sub-acute investigational-product reaction; (ii) the presence of plausible alternative etiologies, including common background gastrointestinal disturbances (dietary variation, transient indigestion, mild dehydration) and non-specific dermatological reactivity; and (iii) the occurrence of a similar gastrointestinal event in the placebo arm, supporting an interpretation that these events may reflect background occurrences rather than a treatment-related effect. However, given the small sample size, a treatment-related contribution cannot be definitively excluded. Severity was graded as moderate for all four events because each prompted symptomatic treatment, although none was clinically significant or required hospitalization.

All hematology, liver-function, renal-function, lipid, and random blood-glucose parameters remained within laboratory reference ranges throughout the study, with no clinically meaningful change within or between arms (Supplementary Tables S3–S5). Vital signs (systolic and diastolic blood pressure, heart rate, pulse rate, respiratory rate, oral temperature) were stable across visits in both arms (all *p* > 0.05).

## 4. Discussion

In this randomized, double-blind, placebo-controlled trial, 28 days of supplementation with 300 mg/day of an *U. lactuca* extract resulted in consistent and statistically significant modulation of multiple systemic biomarkers of oxidative stress and inflammation in healthy adults, alongside a favorable safety profile. The most prominent effects included significant within-group increases in reduced glutathione (GSH) and glutathione peroxidase-1 (GPx-1), concomitant reductions in malondialdehyde (MDA), and significant within-group decreases in circulating CRP, IL-6, IL-11 and IL-1β. These changes were accompanied by an increase in PPAR-γ, a transcriptional regulator with well-established roles in the resolution of inflammation and metabolic homeostasis [25]. To our knowledge, this is the first controlled trial to demonstrate that *U. lactuca* modulates both antioxidant and inflammatory biomarkers simultaneously in healthy, non-obese adults.

The physiological relevance of the observed changes is supported by the coordinated modulation of multiple biomarkers involved in antioxidant defense and inflammatory regulation. The 39.6% increase in GSH, together with the 15.4% increase in GPx-1 and 12.9% increase in catalase, suggests an enhancement of endogenous antioxidant defense capacity and redox homeostasis. Consistent with this interpretation, MDA, a marker of lipid peroxidation, decreased by 18.3%, indicating reduced oxidative damage to cellular lipids. Similarly, reductions in CRP (20.0%), IL-6 (19.0%), and IL-1β (17.4%), accompanied by a 15.2% increase in PPAR-γ, are consistent with a modulation of inflammatory signaling pathways and a shift toward a less pro-inflammatory physiological state. While the present study was not designed to evaluate clinical outcomes, the simultaneous modulation of multiple biomarkers representing complementary biological pathways supports the physiological relevance of the observed responses to *U. lactuca* supplementation.

The magnitude and direction of these changes are consistent with an integrated improvement in systemic redox balance rather than isolated modulation of individual biomarkers. In particular, the simultaneous increase in endogenous antioxidant defenses (GSH, GPx-1, catalase) and reduction in oxidative damage (MDA, thioredoxin) suggest a coordinated shift in redox homeostasis. This pattern mirrors what has been reported in human supplementation trials of other bioactive compounds: oral glutathione supplementation increases erythrocyte and plasma GSH stores over 1–6 months in healthy adults [26], GlyNAC (glycine and *N*-acetylcysteine) supplementation restores glutathione levels in older adults [27], and polyphenol-rich pine bark extract significantly lowers MDA and fibrinogen in healthy older adults over 12 weeks [28]. The present findings extend this paradigm to marine-derived bioactives and suggest that *U. lactuca* may exert comparable systemic effects despite a distinct compositional profile dominated by sulfated polysaccharides rather than polyphenols. Although direct comparisons across studies should be interpreted with caution, the magnitude of the observed biomarker changes is also broadly comparable to those reported for other antioxidant and anti-inflammatory nutraceuticals. For example, coenzyme Q10 supplementation has been associated with improvements in antioxidant status and reductions in circulating inflammatory markers in several populations. Pharmaceutical agents such as statins have likewise been shown to influence oxidative and inflammatory pathways; however, these medications are prescribed for specific clinical indications and exert their effects through mechanisms distinct from those of dietary bioactives.

Mechanistically, these observations align with a growing body of preclinical literature indicating that multiple *U. lactuca*-derived bioactives, including sulfated polysaccharides, lipid-derived electrophiles, and other secondary metabolites, exert pleiotropic effects on oxidative stress and inflammation. Ulvans directly scavenge ROS through electron-donating hydroxyl and sulfate groups [9,12], and low-molecular-weight ulvan oligosaccharides have been shown to attenuate dextran-sulfate-sodium-induced colitis in mice by suppressing TNF-α, IFN-γ, and IL-1β while restoring mucosal barrier integrity [15]. In macrophage models, selenium nanoparticles decorated with *U. lactuca* polysaccharides inhibit NF-κB activation and downstream cytokine release [16]. Beyond direct radical scavenging, ulvans may modulate intracellular redox signaling by up-regulating Phase II antioxidant enzymes via Keap1/Nrf2-dependent and -independent mechanisms [13,14]. In addition to the polysaccharide fraction, the co-extracted lipid components may also contribute to Nrf2 activation. Notably, electrophilic lipid species identified in *Ulva*-derived extracts (e.g., keto-unsaturated C18 fatty acids) have been shown to interact with Keap1 cysteine residues, thereby promoting Nrf2 stabilization and downstream antioxidant gene expression [11]. The increase in PPAR-γ observed here is particularly noteworthy: activation of this nuclear receptor suppresses pro-inflammatory cytokine transcription, promotes M2 macrophage polarization, and improves metabolic regulation [25]. The combined modulation of redox and transcriptional pathways observed in the present study is therefore consistent with a multi-target mechanism of action, rather than a single-pathway pharmacological effect.

Our findings can be interpreted alongside recent clinical studies on Ulva-derived polysaccharides. Roach et al. [18] reported that 6 weeks of SXRG84 supplementation (2–4 g/day) in overweight adults reduced non-HDL cholesterol, CRP, and the atherogenic index; a subsequent crossover trial by the same group [19] demonstrated significant reductions in IFN-γ, IL-1β, TNF-α, and IL-10 at the 2 g/day dose. Allaert et al. [17] earlier demonstrated tolerability and mood-related bioactivity of an edible *U. lactuca* extract in volunteers with anhedonia. It is important to note that SXRG84 represents a purified sulfated polysaccharide fraction, whereas the present study employed a lower-dose, multi-component extract of *U. lactuca* containing not only sulfated polysaccharides but also polyphenols, pigments, and bioactive lipid constituents. Differences in extraction methods, composition, and bioactive concentration limit direct dose comparisons between studies. The present trial extends these observations by providing the first evidence of coordinated antioxidant and anti-inflammatory effects in a healthy, normal-weight population at a lower administered dose (300 mg/day), suggesting that even modest intake of a compositionally complex *U. lactuca* extract may be sufficient to engage these pathways. Broader parallels can be drawn with the marine nutraceutical literature. Brown-algae-derived polyphenols from *Ascophyllum nodosum* modestly reduced DNA damage in obese adults in a crossover trial, although no significant changes in CRP or inflammatory cytokines were observed at the tested dose [29]. Fucoidan, a sulfated polysaccharide from brown algae, has demonstrated immunomodulatory and anti-inflammatory effects in preclinical and early clinical work [30]. The observation that a green-algae-derived sulfated polysaccharide (ulvan) reduced CRP and cytokine levels in healthy adults is therefore noteworthy and positions *U. lactuca* alongside these established marine bioactives.

Several findings warrant cautious interpretation. First, although between-group differences at Day 28 were statistically significant for most biomarkers, catalase and GPx-1 did not reach significance at the single time-point comparison despite significant differences in change-from-baseline. This apparent dissociation likely reflects greater between-subject variability at a single time point and highlights the increased sensitivity of within-subject change analyses in small parallel-group trials [20]. Second, the small but statistically significant increase in CRP in the placebo group is most plausibly explained by normal biological variability, as CRP is known to fluctuate in response to minor physiological and environmental factors [31]; we present this transparently rather than minimizing it. Third, while IL-11 decreased within the active arm, this finding should be interpreted in the context of emerging evidence that identifies IL-11 as a predominantly pro-inflammatory cytokine involved in tissue remodeling and inflammatory signaling. In contrast to earlier views suggesting pleiotropic or protective roles, more recent studies indicate that IL-11 signaling contributes to chronic low-grade inflammation and tissue remodeling [22,23]. However, given the absence of a statistically significant between-group difference and the exploratory nature of the present study in a healthy population with low baseline inflammatory burden, the biological relevance of this observation remains uncertain. Therefore, the observed reduction in IL-11 should be considered hypothesis-generating rather than as evidence of a definitive effect of *U. lactuca* supplementation on IL-11 signaling. Further studies incorporating mechanistic endpoints and populations with elevated inflammatory tone are warranted to clarify whether IL-11 modulation represents a reproducible response to *U. lactuca* supplementation.

The safety profile observed in this study was favorable and consistent with the expected tolerability of marine-derived nutraceuticals. All reported adverse events were mild-to-moderate, transient, and resolved with standard symptomatic care. Causality assessment using the Naranjo scale classified all events as “doubtful”, supported by delayed onset (≥20 days in the active arm; ≥10 days in the placebo arm), lack of a consistent temporal relationship, and the occurrence of a similar gastrointestinal event in the placebo arm [21]. The absence of any clinically meaningful changes in hematological or biochemical safety parameters further supports the tolerability of the intervention at the tested dose. These findings are consistent with the broader safety literature on edible green seaweeds, which have a long history of dietary use and are generally recognized as safe [7,8].

Strengths of this study include the rigorous randomized double-blind design, prospective trial registration, prospectively defined ITT and PP populations, full retention of all randomized participants in the ITT analysis, and the breadth of the biomarker panel evaluated. An additional strength is the inclusion of a healthy, normal-BMI, physically active population, which allows assessment of subtle modulation of oxidative stress and inflammatory pathways in the absence of overt disease or elevated baseline biomarker burden, a context highly relevant to nutritional interventions. The trial also has clear limitations. The sample size (*n* = 10 per arm) is small and was selected on feasibility grounds rather than formal power [20], which restricts both subgroup analysis and the precision of effect-size estimates; some between-group differences that did not reach significance may have been undetected for that reason. While the use of a healthy population strengthens internal validity, it may limit direct generalization to clinical populations with higher baseline oxidative stress or inflammation, in whom larger absolute effects might be expected. Inflammatory biomarkers may be influenced by factors such as recent infection, habitual physical activity, dietary intake, and other lifestyle variables. Although randomization resulted in comparable baseline values between groups, these factors were not comprehensively assessed or standardized and may have contributed to inter-individual variability in the measured outcomes. Intra-assay and inter-assay coefficients of variation were not reported by the analytical laboratory. Although all samples for a given biomarker were analyzed in duplicate within a single analytical run to minimize analytical variability, the absence of formal assay precision metrics represents a methodological limitation. Because the investigational product is a compositionally complex extract, the present study cannot determine the relative contribution of individual constituents to the observed biological responses. Future studies incorporating detailed compositional analyses and fractionation approaches are warranted to identify the specific bioactive components responsible for the observed effects. An additional limitation of this study is the potential influence of sex-related physiological differences on oxidative stress and inflammatory biomarkers. Although no significant baseline differences in sex distribution were observed between groups, hormonal fluctuations associated with the menstrual cycle may affect systemic antioxidant and inflammatory status. Because the study was not designed or powered to evaluate sex-specific responses, and menstrual cycle phase was not assessed, the potential contribution of sex and hormonal status to the observed outcomes cannot be determined. Furthermore, the relatively large number of biomarkers evaluated increases the possibility of Type I error. Because this exploratory pilot study was designed to assess a predefined panel of mechanistically related outcomes, formal adjustment for multiple comparisons was not applied; therefore, the findings should be considered hypothesis-generating and require confirmation in larger adequately powered studies.

The findings of this pilot trial support confirmation in larger cohorts of healthy individuals, with longer intervention periods (e.g., 8–12 weeks) and additional intermediate sampling time points to better characterize the temporal dynamics of antioxidant and inflammatory biomarkers. Dose-ranging studies are warranted to determine whether the effects observed at 300 mg/day are dose-dependent and to define the minimal effective intake for sustained modulation of redox and inflammatory pathways. Further mechanistic work to delineate the relative contributions of ulvan, polyphenol, pigment, and lipid fractions will inform compositional standardization and optimize extract design. Future studies with larger sample sizes should investigate whether responses to *U. lactuca* extract differ between males and females. Finally, the integration of functional outcomes relevant to healthy populations, such as exercise recovery, vascular function, or cognitive performance, alongside biomarker endpoints, would enhance the translational relevance of future studies.

## 5. Conclusions

Daily oral supplementation with 300 mg of an *U. lactuca* extract for 28 days was safe, well tolerated, and produced statistically significant favorable changes across a broad panel of systemic antioxidant and inflammatory biomarkers in healthy adults, including increases in GSH, GPx-1, catalase activity, and PPAR-γ, and decreases in MDA, thioredoxin, CRP, IL-6, IL-11 and IL-1β. These pilot findings provide the first controlled biochemical evidence in humans that *U. lactuca* modulates oxidative-stress and low-grade inflammation pathways simultaneously, and motivate larger, longer-duration, mechanism-oriented trials in populations with elevated baseline biomarker burden.

## Figures and Tables

**Table 1:** Baseline demographic and anthropometric characteristics (ITT population). Values are mean ± SD or *n* (%).

Characteristic	Group ALX (*n* = 10)	Group PLB (*n* = 10)	*p*-Value
Age, years	28.7 ± 4.6	30.8 ± 4.0	0.323 ^a^
Female sex, *n* (%)	6 (60%)	2 (20%)	0.170 ^b^
Weight, kg	64.3 ± 6.6	64.0 ± 7.7	0.791 ^a^
Height, cm	165.7 ± 7.4	166.00 ± 6.6	0.924 ^c^
BMI, kg/m^2^	23.4 ± 0.8	23.14 ± 1.2	0.639 ^c^

^a^ Mann–Whitney U test; ^b^ Fisher’s exact test; ^c^ independent-samples *t*-test.

**Table 2:** Within- and between-group changes in antioxidant and inflammatory biomarkers from Day 0 to Day 28 (ITT population, *n* = 10 per arm).

Biomarker (Unit)	Group	Day 0 (Mean ± SD)	Day 28 (Mean ± SD)	Δ Day 0–28	Between-Group *p* (Δ; Day 28)
**GSH (ng/mL)**	ALX	10.1 ± 1.3	14.1 ± 1.9	+4.0 ± 1.9 ***	<0.0001/<0.0001
	PLB	10.6 ± 1.4	10.0 ± 1.2	−0.6 ± 0.6 **	
**MDA (nmol/mL)**	ALX	33.4 ± 5.7	27.4 ± 4.8	−6.1 ± 2.9 ***	<0.0001/0.032
	PLB	33.3 ± 6.4	33.2 ± 6.2	−0.1 ± 1.0	
**Catalase (kU/L)**	ALX	250.3 ± 59.5	282.7 ± 59.7	+32.4 ± 22.2 **	0.001/0.174
	PLB	251.0 ± 32.5	251.9 ± 32.5	+0.9 ± 3.5	
**GPx-1 (µU/mL)**	ALX	440.3 ± 79.9	508.1 ± 95.7	+67.8 ± 27.7 ***	<0.001/0.083
	PLB	440.9 ± 69.9	437.8 ± 73.3	−3.0 ± 7.7	
**Thioredoxin (ng/mL)**	ALX	11.5 ± 1.0	10.0 ± 1.3	−1.5 ± 0.7 **	0.002/0.007
	PLB	11.8 ± 1.1	11.4 ± 0.9	−0.4 ± 0.7	
**CRP (mg/L)**	ALX	4.5 ± 0.8	3.6 ± 0.7	−0.9 ± 0.4 ***	<0.0001/0.014
	PLB	4.4 ± 0.6	4.4 ± 0.6	+0.1 ± 0.1 *	
**IL-6 (pg/mL)**	ALX	15.8 ± 1.3	12.8 ± 1.5	−3.0 ± 1.1 ***	0.002/0.0002
	PLB	15.8 ± 1.3	15.7 ± 1.2	−0.1 ± 0.2	
**IL-1β (pg/mL)**	ALX	51.1 ± 6.0	42.2 ± 4.0	−8.9 ± 3.6 ***	<0.001/0.0007
	PLB	51.0 ± 5.3	51.1 ± 5.4	+0.1 ± 0.4	
**IL-11 (ng/mL)**	ALX	58.7 ± 7.6	54.4 ± 7.7	−4.3 ± 1.9 ***	<0.0001/0.193
	PLB	58.8 ± 6.6	58.7 ± 6.4	−0.1 ± 0.9	
**PPAR-γ (ng/L)**	ALX	37.5 ± 4.7	43.1 ± 5.5	+5.7 ± 2.3 **	0.001/0.007
	PLB	37.5 ± 3.9	37.6 ± 4.3	+0.2 ± 0.5	

ALX = *U. lactuca* extract; PLB = placebo; Δ = mean change. Within-group (paired) significance: *** *p* < 0.001; ** *p* < 0.01; * *p* < 0.05. Between-group *p*-values are presented as *p* (change from baseline)/*p* (Day 28).

**Table 3:** Adverse events reported during the 28-day treatment period.

Visit	Group	Adverse Event (Preferred Term)	System Organ Class	Onset/Resolution	Symptomatic Treatment
2	PLB	Bloating	Gastrointestinal	9 December 2025 → 11 December 2025	Simethicone 140 mg
3	ALX	Skin rash	Skin and subcutaneous tissue	18 December 2025 → 21 December 2025	Cetirizine 10 mg
3	ALX	Nausea	Gastrointestinal	26 December 2025 → 28 December 2025	Ondansetron 4 mg
3	ALX	Mild diarrhoea	Gastrointestinal	31 December 2025 → 2 January 2026	Oral rehydration solution

## Data Availability

Data and statistical analyses are available for non-commercial scientific inquiry and/or educational use if request and use do not violate IRB restrictions and/or research agreement terms.

## Supplementary Materials

The following supporting information can be downloaded at: https://www.mdpi.com/article/10.3390/nu18142244/s1, Table S1: Per-protocol antioxidant biomarkers (*n* = 9/arm); Table S2: Per-protocol inflammatory biomarkers (*n* = 9/arm); Table S3: Hematology at Screening, Day 14, Day 28 (ITT, *n* = 10/arm); Table S4: Biochemistry at Screening, Day 14, Day 28 (ITT, *n* = 10/arm); Table S5: Vital signs at Day 0, Day 14, Day 28 (ITT, *n* = 10/arm).
